# Colon interposition after esophagectomy: a 15-year single-center experience

**DOI:** 10.1007/s00423-026-04140-0

**Published:** 2026-07-25

**Authors:** Melissa Kemeter, Lucas Thumfart, Patrick Heger, Felix J. Hüttner, Markus K. Diener, Luca Giulini, Attila Dubecz

**Affiliations:** Department of Surgery, Paracelsus Medical University Nuremberg, Nuremberg, Germany

**Keywords:** Esophagectomy, Colon Interposition, Esophageal Cancer, Reconstruction

## Abstract

**Background:**

Colonic interposition is the preferred option for alimentary tract reconstruction when a gastric conduit pull-up is not feasible. Aim of this study was to evaluate perioperative outcomes of patients undergoing colonic interposition for various indications in our tertiary-care center over a 15-year period.

**Methods:**

Following institutional board approval, all consecutive patients who underwent colonic interposition for esophageal replacement were identified from a prospectively maintained database. Comprehensive chart review and descriptive statistical analyses were performed.

**Results:**

Ninety-three patients (62 men, 31 women, median age 65 years, IQR 55.5–72) underwent colonic interposition between January 2009 and December 2023. Benign disease was present in 17.2%, whereas malignancy accounted for 82.8%. A hand-sewn cervical anastomosis was performed in 96.8% of patients. The colonic graft, predominantly based on the left colic artery (89.2%) was routed through the posterior mediastinum in 51.6% and via the retrosternal route in 47.3%. Median operative time was 303 min (IQR 247.5–364). Major complications included anastomotic leakage (28%), anastomotic stenosis (30.1%), pleural empyema (11.8%), and postoperative bleeding (10.8%). The reoperation rate was 38.7%, with a median hospital stay of 29 days (IQR 20–63). In-hospital mortality was 18.3%.

**Conclusion:**

Colonic interposition after esophagectomy is associated with substantial morbidity and mortality, particularly in the salvage and secondary reconstruction setting, and should be confined to experienced, high-volume centers.

**Clinical registration number:**

FMS_D_007.23-I-1

## Introduction

Esophagectomy remains one of the most technically demanding procedures in gastrointestinal surgery, associated with considerable morbidity and mortality rates [1]. 

Gastrointestinal reconstruction after esophageal resection is most commonly performed using a gastric conduit, favored for its reliable vascularization, ease of mobilization, and acceptable morbidity profile [2]. 

When the stomach is unavailable or unsuitable – for example, after previous gastric surgery, when simultaneous gastrectomy is required, or when the gastroepiploic arcade is interrupted or injured – colonic interposition becomes the preferred alternative [3]. 

The first successful colonic interpositions were described in 1911 by Kelling and Vuillet, who used a segment of colon to replace the esophagus in a patient with caustic injury, achieving restoration of oral intake following retrosternal transposition and primary pharyngeal anastomosis [4, 5]. 

In 1956, Sherman and Waterston adapted the technique for children with esophageal atresia [6]. Since then, colonic interposition has been used for both benign and malignant esophageal diseases [7–12]. 

Although minimally invasive approaches are now standard for both colorectal and complex upper gastrointestinal (GI) surgery [13–16], colonic interposition remains a highly invasive, technically demanding procedure performed predominantly in specialized high-volume centers [11, 17–19]. 

Reports on minimally invasive colonic mobilization for esophageal replacement remain scarce, largely due to the operation’s complexity and the requirement for extensive multidisciplinary expertise [20]. 

Moreover debate persists regarding the optimal surgical technique, particularly concerning vascular supply and route of reconstruction [21–26]. 

Most centers favor a left colic artery-based graft combined with retrosternal pull-up, as this approach is associated with lower morbidity and favorable long-term functional outcomes [27]. 

The aim of this study was to analyze perioperative outcomes of all patients who underwent colonic interposition for various indications at our tertiary-care center over a 15-year period.

## Methods

After obtaining institutional review board (IRB) approval (IRB No.: FMS_D_007.23-I-1 Date of approval: 12/1/2023), all patients who underwent colonic interposition for esophageal replacement at Klinikum Nürnberg Nord (Germany) between January 2009 and December 2023 were identified from a prospectively maintained institutional database.

A comprehensive chart review was conducted to verify and complete any missing data on patient’s demographics, perioperative parameters and clinical outcomes. Following variables were collected and analyzed: preoperative data (age, sex, ASA classification, body mass index (BMI), tumor characteristics, indication for surgery), intraoperative details (operation time, surgical technique, graft type, vascular supply, route of reconstruction, anastomotic technique), and postoperative outcomes (complications, reoperation rate, mortality, cause of death, length of stay). Primary reconstruction was defined as reconstruction using a colonic interposition performed during the initial surgical procedure, for example in cases requiring simultaneous gastrectomy. Secondary reconstruction was defined as reconstruction with a colonic interposition following complications of a gastric pull-up, recurrence of cancer within the gastric conduit, or the need to restore gastrointestinal continuity.

Major complications were defined as those classified as Clavien–Dindo grade IIIb or higher.

Data analysis was performed using descriptive statistics. Continuous variables were reported as medians with interquartile ranges (IQR), while categorical data were presented as frequencies and percentages.

## Results

### Patient characteristics

A total of 93 patients underwent colonic interposition during the study period, comprising 62 men and 31 women. The median age was 65 years (IQR range 55.5–72).

Colon interposition was performed for benign disease in 16 (17.2%) and for malignant disease in 77 (82.8%) patients. Among cancer patients, adenocarcinoma represented the most frequent histological subtype (*n* = 52, 67.5%). The most common tumor locations were the distal esophagus (*n* = 21; 22.6%) and the esophagogastric junction (EGJ) (*n* = 20; 21.5%).

Neoadjuvant treatment was administered in 28 cancer patients (30.1%), including radiochemotherapy in 12, chemotherapy alone in 15 and radiotherapy alone in 1 patient.

Most patients were classified as ASA III (*n* = 52; 55.9%), followed by ASA II (*n* = 35; 37.6%) and ASA IV (*n* = 6; 6.5%).

The median preoperative body mass index (BMI) was 22.1 kg/m^2^ (IQR range 19.8–25.6).

In most patients (*n* = 50; 53.8%) colon interposition was used as a secondary reconstruction.

Discontinuity of the gastrointestinal tract after previous esophagectomy with complicated course (e.g. necrosis of the conduit, anastomotic leakage) was the most common indication (*n* = 32; 34.4%) in that group. Resection of the gastric conduit due to recurrent carcinoma (*n* = 12; 12.9%) or postoperative complications after esophagectomy with a gastric conduit during with immediate reconstruction (*n* = 6; 6.5%) represented additional indications for secondary reconstruction. Simultaneous gastrectomy (*n* = 31; 33.3%) was the most frequent indication for primary colonic interposition, performed in 25 patients (26.9%) due to tumor size or oncologic margins and in 6 patients (6.5%) due to intraoperative complications such as gastric conduit hypoperfusion. The various indications for colonic interposition are summarized in Table 1.

**Table 1:** Indications for colon interposition

Variable	Number *N* (%)
Primary Reconstruction	**43 (46.2%)**
Primary	10 (10.7%)
Simultaneous gastrectomy	31 (33.3%)
- Due to tumor size/ oncologic margins	25 (26.9%)
- Due to intraoperative complications	6 (6.5%)
Gastric surgery in the past	2 (2.2%)
Secondary Reconstruction	**50 (53.8%)**
Recurrent Cancer	12 (12.9%)
Postoperative complications	6 (6.5%)
Discontinuity	32 (34.4%)
- Previous simultaenous gastrectomy	12 (12.9%)
- Previous resection of gastric conduit (Ischemia, anastomosic leakage)	14 (15.1%)
- Stomach left in place	6 (6.5%)

## Intraoperative data

Median operative time was 303 min (IQR range: 247.5–364). Most procedures (*n* = 84; 90.3%) were performed via open surgery. In nine patients, surgery was initiated laparoscopically or robotically assisted but required conversion to laparotomy due to intraoperative complications such as bleeding or inadequate oncologic resection margins.

For reconstruction, the left (*n* = 1; 1.1%) or transverse colon (*n* = 82; 88.2%), pedicled on the ascending branch of the left colic artery (inferior mesenteric artery territory), was used in 83 patients (89.2%); a right graft based on the middle colic artery (superior mesenteric artery territory) was used in 10 cases (10.8%). No graft was based on the right colic artery. For left colic-based grafts the middle colic vessels were systematically ligated, with perfusion maintained through the marginal artery of Drummond.

Regarding the reconstruction route, the posterior mediastinum was chosen in 48 (51.6%), the retrosternal space in 44 (47.3%), and the intrapleural (left parasternal) cavity in one patient (1.1%) who had previously undergone sternotomy.

A hand-sewn cervical anastomosis was performed in 90 patients (96.8%). A feeding jejunostomy was placed in 86 cases (92.5%). Alimentary continuity was restored by colojejunostomy (Billroth II) and ileo-colostomy in all patients.

A summary of intraoperative details is provided in Table 2.

**Table 2:** Summary of intraoperative data

Variable	number *n* (%)
Graft	
Transverse colon	82 (88.2%)
Right colon	10 (10.8%)
Left colon	1 (1.1%)
Graft’s blood supply	
Left colic artery	83 (89.2%)
Middle colic artery	10 (10.8%)
Reconstruction route	
Posterior mediastinum	48 (51.6%)
Retrosternal	44 (47.3%)
Intrapleural	1 (1.1%)
Anastomosis	
Cervical anastomosis	90 (96.8%)
Thoracic anastomosis	3 (3.2%)

## Postoperative morbidity

Overall postoperative morbidity (any Clavien-Dindo grade) was 96.8% (*n* = 90) [28]. 

Only three patients (3.2%) had an uneventful postoperative course. Twenty-six patients (28%) experienced CD Grade II complications, while 52 (55.9%) experienced major complications (Clavien-Dindo ≥ IIIb). Grade III complications occurred in 29 (31.2%), including 12 patients (12.9%) with grade IIIa and 17 patients (18.3%) with grade IIIb complications. Eighteen patients (19.4%) developed grade IV complications (13 patients [13.9%] grade IVa, 5 patients [5.4%] grade IVb). Seventeen (18.3%) patients died (grade V).

**Fig. 1:**
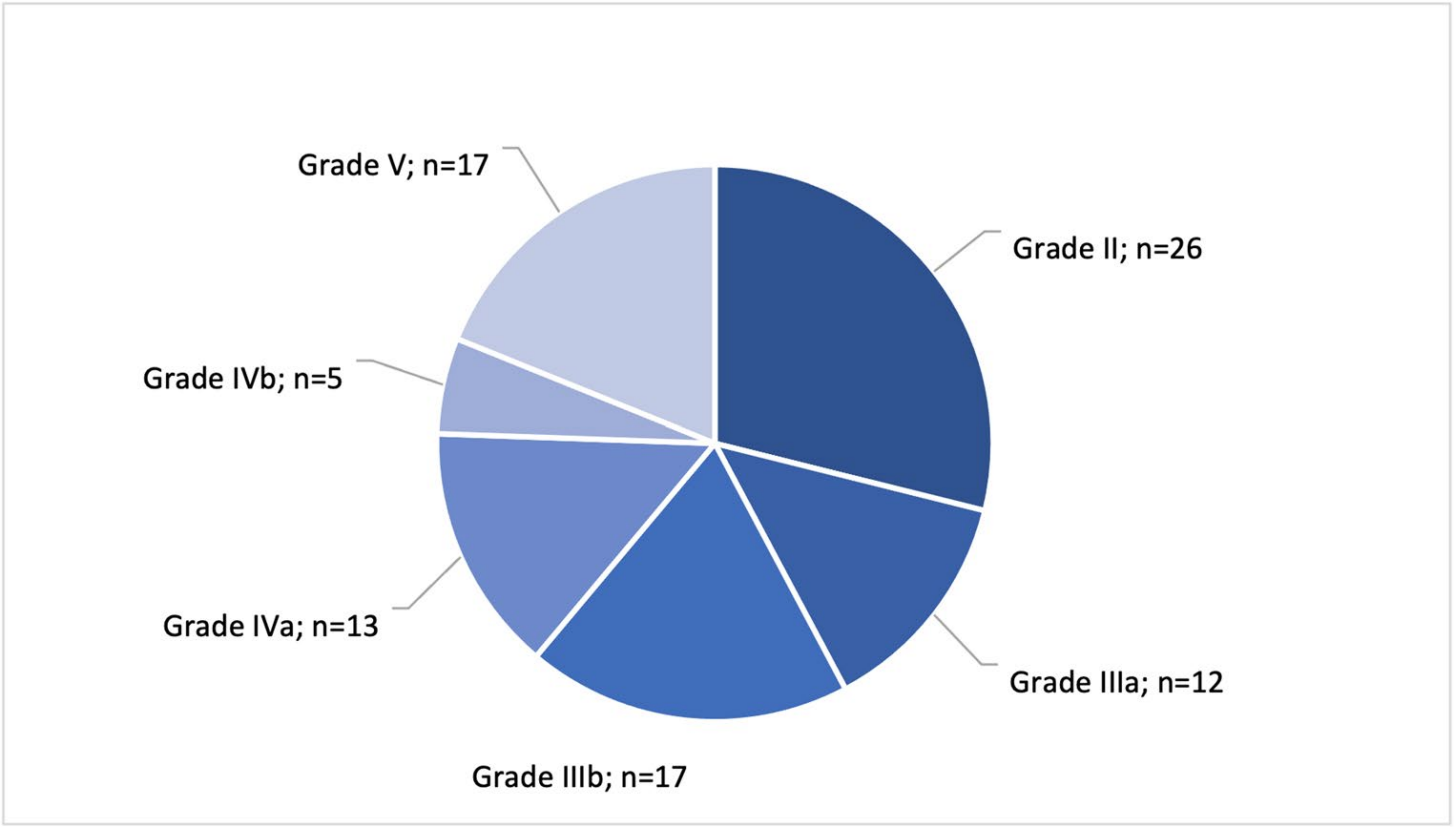
Pie chart showing the distribution of perioperative morbidity according to the Clavien-Dindo Classification

Figure 1 is showing the distribution of perioperative morbidity according to the Clavien-Dindo classification.

The most frequent surgical complication was anastomotic stenosis (*n* = 28; 30.1%), requiring repeated endoscopic dilations. Anastomotic leakage or other insufficiencies (e.g. duodenal or gastric) occurred in 26 patients (28%), with early leaks (< 5 days postoperatively) diagnosed in 9 patients (9.7%). 17 out of the 26 patients demonstrated a leakage in the cervical anastomosis, while 7 patients had an abdominal leakage either at anastomotic or duodenal stump level. 2 patients (2.2%) had both a cervical and an abdominal leak. There was no anastomotic leakage in the 3 patients (3.2%) who had a thoracic anastomosis.

Additional complications are summarized in Table 3.

**Table 3:** Postoperative complications

Variable	number *n* (%)
**Surgical complications**	
Anastomotic stenosis	28 (30.1%)
Anastomotic leakage	26 (28%)
- Cervical anastomosis	17 (20.4%)
- Abdominal anastomosis or duodenal/gastric insufficiency	7 (7.5%)
- Cervical and abdominal anastomosis	2 (2.2%)
Wound infection	24 (25.8%)
Pleural empyema	11 (11.8%)
Postoperative bleeding	10 (10.8%)
Necrosis/ ischemia of the colon	4 (4.3%)
Injury of recurrent laryngeal nerve	2 (2.2%)
Injury to the bronchial system	2 (2.2%)
Chylothorax	1 (1.1%)
**Non- Surgical complications**	
Pneumonia	40 (43%)
Sepsis	20 (21.5%)
Arrhythmia	26 (28%)
Pulmonary embolism	2 (2.2%)

**Table 4:** Selected surgical complications according to colon graft, blood supply of the graft, reconstruction route and location of the anastomosis

	Complication		
	Anastomotic Leakage (*n = 26; 28%)*	Anastomotic Stenosis (*n = 28; 30.1%)*	Ischemia (*n = 4; 4.3%)*
**Colon graft**			
Right colon	1 (1.1%)	2 (2.2%)	0 (0%)
Transverse colon	25 (26.9%)	26 (28%)	4 (4.3%)
Left colon	0 (0%)	0 (0%)	0 (0%)
**Blood supply**			
Left colic artery	25 (26.9%)	26 (28%)	4 (4.3%)
Middle colic artery	1 (1.1%)	2 (2.2%)	0 (0%)
**Reconstruction**			
Posterior mediastinum	12 (12.9%)	10 (10.6%)	2 (2.2%)
Retrosternal	13 (14%)	18 (19.4%)	2 (2.2%)
Intrapleural	1 (1.1%)	0 (0%)	0 (0%)
**Anastomosis**			
Cervical	17 (18.3%)	28 (30.1%)	4 (4.3%)
Abdominal	7 (7.5%)	0 (0%)	0 (0%)
Both (cervical and abdominal)	2 (2.2%)		

Table 4 presents rates of anastomotic leakage, anastomotic stenosis, and conduit ischemia stratified by conduit type, vascular supply, reconstruction route, and anastomotic location.

The overall reoperation rate was 38.7% (*n* = 36), with 20 patients (21.5%) undergoing more than one surgical intervention. Besides surgical complications, other indications for reoperation included perforated diverticulum (*n* = 2; 2.2%), bile leakage (*n* = 3; 3.2%), hematoma or abscess evacuation (*n* = 7; 7.5%), and intestinal ischemia (*n* = 1; 1.1%). Pulmonary complications were the most common non- surgical issues, affecting 40 patients (43%), including pneumonia, prolonged ventilation, or the need for bronchoscopic interventions. Nine patients (9.7%) underwent tracheostomy and 20 patients (21.5%) developed sepsis. Blood transfusions were required in 79 patients (84.9%) at some point during hospitalization.

## Postoperative mortality

In-hospital mortality was 18.3%. The leading cause of death was septic multiorgan failure, most frequently associated with anastomotic leakage or pneumonia. One patient died intraoperatively from hemorrhage and ventricular tachycardia during revision surgery.

The most common causes of death are summarized in Table 5.

**Table 5:** Causes of in hospital-death

Variable	Number *n* (%)
Septic multi organ failure	10 (10.8%)
- Due to respiratory failure due to pneumonia	2 (2.2%)
- Due to anastomotic leakage/ necrosis	4 (4.3%)
- Due to intestinal ischemia	1 (1.1%)
- Due to septic cardiomyopathie	1 (1.1%)
- Due to bronchial fistula	1 (1.1%)
- Due to infected hematoma (thoracic and abdominal)	1 (1.1%)
Cardiac arrest/ Asystole	2 (2.2%)
Respiratory failure (palliative setting)	1 (1.1%)
Pulmonary embolism	1 (1.1%)
Stroke	1 (1.1%)
Hemorrhagic shock	1 (1.1%)
Intraoperative ventricular tachycardia	1 (1.1%)

**Table 6:** Mortality according to colon graft, blood supply, reconstruction route and location of anastomosis, primary vs. secondary reconstruction and indication (benign/malignant)

**Colon graft**		
Right colon		1/10 (10%)
Transverse colon		16/82 (19.5%)
Left colon		0 (0%)
**Blood supply**		
Left colic artery		16/83 (19.3%)
Middle colic artery		1/10 (10%)
**Reconstruction**		
Posterior mediastinum		11/48 (22.9%)
Retrosternal		6/44 (13.6%)
Intrapleural		0 (0%)
**Anastomosis**		
Cervical		17/90 (18.9%)
Thoracic		0 (0%)
**Primary** **vs** **Secondary Reconstruction**		
Primary reconstruction		7/43 (16.3%)
Secondary reconstruction		10/50 (20%)
**Indication**		
Benign		1/16 (6.3%)
Malignant		16/77 (20.8%)

Stratified in-hospital mortality by indication, reconstruction type, conduit, vascular pedicle, and route is shown in Table 6. Given the small number of events (*n* = 17), these comparisons are descriptive only; formal significance testing was not performed, as it would be underpowered.

### Length of hospital stay

Median hospital stay was 29 days (IQR range 20–63). Patients spent a median of 12 days in the surgical ICU (IQR range 6.5–30.5).

## Discussion

This study presents a 15-year single center experience with colonic interposition after esophagectomy and represents one of the largest reported European series to date. Most previously published single-center cohorts have included between 19 and 90 patients [11, 18, 29, 30], whereas our 93-patient cohort provides a more robust dataset for analyzing perioperative outcomes, complications, and technical considerations.

In our 15-year single-center series, morbidity and mortality remained within these ranges, despite advances in perioperative care, reflecting both the complexity of the patient population and the inherent technical demands of the procedure. The literature describes colonic interposition as an essential reconstructive option when gastric or jejunal conduits are unavailable, such as after prior gastric resection, simultaneous gastrectomy, or ischemic complications of the gastric conduit [3, 12, 22]. 

Our findings align with these indications, as the majority of our procedures were performed for discontinuity after esophagectomy, salvage reconstruction, or primary reconstruction requiring simultaneous gastrectomy, highlighting the heterogeneous and complex nature of this patient population.

Recent years have seen growing interest in minimally invasive techniques for esophageal and colorectal surgery, with several centers reporting encouraging early results [13–16]. 

However, even in these studies, conversion rates remain substantial, and outcomes have not consistently matched those of open surgery. In our cohort, all laparoscopic or robotic attempts required conversion to open surgery, confirming the current limitations of minimally invasive approaches in colonic interposition.

Meta-analyses have shown that left colic artery-based grafts are associated with lower morbidity, mortality, and anastomotic leak rates compared with right-sided grafts, largely due to the more consistent vascular anatomy of the left colon [23, 27, 31, 32]. 

Our practice reflects these findings, as we predominantly used left colic artery-based grafts, reserving right-sided grafts for selected cases where anatomy or oncologic factors required it. The posterior mediastinal route replicates the native esophageal trajectory and has been associated with lower anastomotic tension and potentially lower leak rates in several studies [17, 21, 25, 33]. However, mediastinal sepsis after leakage remains a major concern [34, 35]. 

Although the majority of anastomotic leakage involved the cervical anastomosis and was drained externally, a leak along a conduit placed in the posterior mediastinum may track caudally and result in mediastinitis. Intrathoracic anastomosis (3.2% in our cohort) could carry the highest risk of contained mediastinal sepsis.

The retrosternal route, recommended in cases with mediastinal adhesions or previous thoracic surgery [36], has also been associated with favorable outcomes in meta-analyses compared to the posterior mediastinal route [27]. In our cohort, cervical anastomotic leakage occurred at comparable rates in both routes (posterior mediastinum 10/48, 20.8%; retrosternal 8/44, 18.2%), with no clinically meaningful difference between approaches.

Our center used both approaches selectively with outcomes comparable to those reported in the literature, supporting individualized route selection based on anatomy and prior surgical history. Multiple meta-analyses and prospective trials have confirmed higher leak rates with cervical compared to intrathoracic anastomoses, though cervical leaks are often easier to manage and associated with lower mortality [37, 38]. 

Our findings parallel these reports: while cervical anastomoses had a higher leak and stricture rate, early detection protocols and local management strategies allowed effective treatment in most cases. Throughout the study period, all cervical anastomoses were hand-sewn. We have since adopted motorized circular staplers for cervical reconstruction, reflecting trends in intrathoracic surgery where stapling has been associated with reduced leak rates [39]; as these procedures were performed after the study period, they are not represented in the present cohort and no comparative subanalysis was possible.

Several studies have documented improvements in the management of postoperative complications, particularly anastomotic leakage with the introduction of self-expanding metal stents and, more recently, endoluminal vacuum therapy [40]. 

Similar to these reports, our experience confirms that early endoscopic interventions have reduced the need for external cervical fistulas and improved overall outcomes after leakage.

Our overall mortality exceeded the sub-10% rates reported from high-volume centers [27]. 

This likely reflects the composition of our cohort, which was predominantly malignant (82.8%) and weighted toward secondary and salvage reconstructions after failed gastric conduits, recurrent malignancy, or multiple prior operations, a substantially higher-risk population than that of series reporting lower mortality. Larger malignant series have likewise reported higher morbidity and mortality than benign cohorts [30, 41]. 

Long-term functional and quality-of-life results after colonic interposition have generally been favorable in published series [8, 42]. 

Systematic assessment of quality of life was not feasible in this retrospective study, primarily because long-term follow-up data were unavailable for a proportion of patients who were either lost to follow-up or had died before data collection.

Robotic platforms, particularly single-port systems, have demonstrated feasibility for colonic mobilization in colorectal surgery and are increasingly being explored in esophageal reconstruction [43, 44]. 

Based on our experience and the available literature, robotic may offer a pathway to reducing invasiveness without compromising outcomes, although long-term data remain limited.

The retrospective single-center design and in particular the lack of systematic quality-of-life assessment limit the generalizability of our findings. Nevertheless, the strengths of this study include a large cohort size, consistent surgical techniques, and detailed perioperative analysis over a 15-year period, providing a comprehensive overview of this complex procedure.

## Conclusion

Colonic interposition after esophagectomy is associated with substantial morbidity and mortality, particularly in the salvage and secondary reconstruction setting, and should be confined to experienced, high-volume centers. Continued improvements in perioperative care, surgical techniques, and emerging technologies such as robotic platforms may help to further enhance safety and long-term results in the future.

## Data Availability

No datasets were generated or analysed during the current study.
